# Mycotoxin Dietary Exposure Assessment through Fruit Juices Consumption in Children and Adult Population

**DOI:** 10.3390/toxins11120684

**Published:** 2019-11-22

**Authors:** Noelia Pallarés, Dionisia Carballo, Emilia Ferrer, Mónica Fernández-Franzón, Houda Berrada

**Affiliations:** 1 Laboratory of Toxicology and Food Chemistry, Faculty of Pharmacy, University of Valencia, Burjassot, 46100 Valencia, Spainemilia.ferrer@uv.es (E.F.);; 2 Faculty of Agricultural Science, National University of Asunción, San Lorenzo 2160, Paraguay

**Keywords:** mycotoxins, fruit juice, DLLME, risk assessment

## Abstract

Consumption of fruit juice is becoming trendy for consumers seeking freshness and high vitamin and low caloric intake. Mycotoxigenic moulds may infect fruits during crop growth, harvest, and storage leading to mycotoxin production. Many mycotoxins are resistant to food processing, which make their presence in the final juice product very likely expected. In this way, the presence of 30 mycotoxins including aflatoxin B1 (AFB1), aflatoxin B2 (AFB2), aflatoxin G1 (AFG1), aflatoxin G2 (AFG2), alternariol (AOH), alternariol monomethyl ether (AME), Ochratoxin A (OTA), fumonisin B1 (FB1), fumonisin B2 (FB2), enniatin A (ENNA), enniatin A1 (ENNA1), enniatin B (ENNB), enniatin B1 (ENNB1), beauvericin (BEA), sterigmatocystin (STG), zearalenone (ZEA), α-zearalanol (α-ZAL), β-zearalanol (β-ZAL), α-zearalenol (α-ZOL), β-zearalenol (β-ZOL), deoxynivalenol (DON), 3-acetyl-deoxynivalenol (3-ADON), 15-acetyl-deoxynivalenol (15-ADON), diacetoxyscirpenol (DAS), nivalenol (NIV), fusarenon-X (FUS-X), neosolaniol (NEO), patulin (PAT), T-2 toxin and HT-2 toxin was evaluated in 80 juice samples collected from Valencia retail Market. An efficient Dispersive Liquid-Liquid Microextraction method (DLLME) was carried out before their trace level determination by chromatographic techniques coupled to tandem mass spectrometry. The results obtained revealed the presence of nine mycotoxins namely AOH, AME, PAT, OTA, AFB1, AFB2, AFG2, β-ZAL, and HT2 in the analyzed samples, with incidences ranging from 3 to 29% and mean contents between 0.14 and 59.52 µg/L. Considerable percentages of TDIs were reached by children when 200 mL was considered as daily fruit juice intake.

## 1. Introduction

Fruit juices are excellent source of antioxidants, vitamins, and minerals, which play an important role in the prevention of heart diseases, cancer, and diabetes. The consumption of fruit juices is trendy nowadays, to meet the goals of five daily serving of fruits and vegetables. Fruit juices are mainly consumed by children and seekers of higher-quality diets due to their freshness, high vitamin content, and low caloric intake [1].

Moulds can infect agricultural crops during growth, harvest, storage, and processing. The fungal growth and the further mycotoxin production in fruits depend on various environmental factors such as moisture content, temperature and pH. Water activity of fruits boost fungal growth as many of them still tolerate the acidic pH of natural acids as citric, malic and tartaric acids [2].

Furthermore, some environmental conditions such as insect infestation, drought, cultivar susceptibility, mechanical damage, rainfall or humidity can promote the mycotoxins production in crops. *Alternaria* spp., *Aspergillus* spp., *Fusarium* spp., and *Penicillum* spp. are the most common postharvest and storage fungi of fruits [1].

Aflatoxins (AFs), ochratoxin A (OTA), patulin (PAT) and the *Alternaria* toxins such as alternariol (AOH), alternariol methyl ether (AME) and altenuene (ALT) are the most common mycotoxins reported in fruits and their processed products [3,4,5,6,7,8,9].

Mycotoxins, secondary fungi metabolites are related to different health adverse effects, such as cancer induction, mutagenicity, estrogenicity as well as gastrointestinal, urogenital, vascular, kidney and nervous disorders [10]. Mycotoxins showed stability against heat processes, which make its occurrence in processed juice highly likely expected [11]. A careful selection of fruits, a proper storage, physical, chemical and biological detoxification methods are some strategies that are adopted to reduce the presence of mycotoxins in fruits [1,2].

The European Union Commission has set permissible maximum limits to control the contents of mycotoxins in fruit juices [12]. For OTA, 2 µg/kg have been established as maximum limit in grape juice, grape nectar, and its concentrated. In the case of PAT, 50 µg/kg have been set in fruit juices, its concentrated and fruits nectars while 10 µg/kg is the maximum established for apple juice and solid apple products, including apple compote and apple puree used by infants and young children. In the case of AFs, maximum limits have been established in dried fruits, but no AFs limits were set in fruit juices. *Alternaria* mycotoxins have been not regulated yet, despite some authors reported the toxicological implications of their presence in fruit juices [13].

Although patulin is the most studied mycotoxin in fruits and their processed products, the presence of *Alternaria* toxins, aflatoxins and ochratoxin A has also been reported [1]. These compounds can be of concern for human health due to their toxicity and the high consumption of juices by the young population, so multimycotoxin occurrence study in different types of juice available in the market will contribute to more realistic risk estimation.

Most methods for mycotoxins extraction involved liquid-liquid extraction, solid phase extraction using C18 cartridges and immunoaffinity clean-up columns to pre-concentrate and purify mycotoxins in food matrices. A dispersive liquid-liquid microextraction (DLLME) has also been developed for the purpose of miniaturizing, simplifying and automating the analytical procedure with good results [14]. DLLME is a ternary component system formed by an aqueous solution, an organic extraction solvent (frequent solvent with high density) and a dispersive solvent (miscible in both of extractant and aqueous phases). DLLME has already proved some advantages such as high recoveries and low-cost applications [9,15,16].

The aim of the present study was to evaluate the presence of 30 mycotoxins (AFB1, AFB2, AFG1, AFG2, AOH, AME, OTA, FB1, FB2, ENNA, ENNA1, ENNB, ENNB1, BEA, STG, ZEA, α-ZAL, β-ZAL, α-ZOL, β-ZOL, DON, 3-ADON, 15-ADON, DAS, NIV, FUS-X, NEO, PAT,T-2 and HT-2 toxins) in 80 fruit juice samples by Dispersive Liquid-Liquid Microextraction method (DLLME) and gas and liquid chromatography-tandem mass spectrometry determination. Liquid chromatography coupled to tandem mass spectrometry was used for the analysis of AFB1, AFB2, AFG1, AFG2, AOH, AME, OTA, FB1, FB2, ENNA, ENNA1, ENNB, ENNB1, BEA, STG while gas chromatography coupled to tandem mass spectrometry was used for the analysis of ZON, α-ZAL, β-ZAL, α-ZOL, β-ZOL, DON, 3-ADON, 15-ADON, DAS, NIV, FUS-X, NEO, PAT,T-2 and HT-2. Furthermore, the exposure to mycotoxins in adults and children population through the consumption of fruit juices was evaluated.

## 2. Results and Discussion

### 2.1. Method Validation

The method was validated in terms of recoveries, repeatability (intra-day precision), intermediate precision (inter-day precision), matrix effects, limits of detection (LODs), limits of quantification (LOQs) and linearity according to European Commission [17]. The results are shown in Table 1.

The acquisition by the triple quadrupole detector of two SRM transitions per compound accomplished the requirements set by the Commission Decision 2002/657/EC as regards ion transition ratio criteria and procedures for the validation of analytical methods satisfied by MS/MS.

Blank apple juice samples were tested for interference and selectivity. Matrix effects (SSE) that evaluate a possible suppression or enhancement of the signal was obtained comparing the slope of mycotoxins calibration curves prepared by spiking blank apple juice samples extract with the slope of calibration curves prepared in methanol. SSE (%) were calculated as follows: SSE (%) = 100× slope of curve in extracted matrix/slope of curve in methanol.

Matrix matched standards were used to compensate the signal suppression/enhancement (SSE) of matrix effects. Evidence for relative matrix effects have been registered for mycotoxins in apple juice as signal suppression (from 41–64%) was observed for α-ZOL, AFB1, AFG1, AFG2, OTA, ENNA1, ENNB1, and BEA. Therefore, matrix-matched calibration curves were used for effective quantification of samples. Matrix-matched calibration curves were prepared by spiking blank juice samples at concentrations between LOQs and 1000 μg/L.

The recoveries were obtained by spiking blank apple juice samples before and after the extraction procedure with the studied mycotoxins at three concentration levels (50, 100 and 200 µg/L) in three replicates. The values obtained ranged from 61 to 115%. To obtain Intra-day precision, three determinations were performed in the same day and in three of non-consecutive days for Inter-day precision. The intra-day and inter-day precision were lower than 14% and 19%, respectively, for all studied mycotoxins.

The LODs and LOQs were determined spiking blank apple juice samples, at decreasing concentrations of analyzed mycotoxins using the criterion for both transitions predetermined per each mycotoxin of S/N ≥ 3 for calculate LOD and S/N ≥ 10 for LOQ. LODs obtained ranged from 0.15 to 2.34 μg/L and the LOQs from 0.5 to 7.81 μg/L. The relative error between prepared concentrations and the obtained ones ranged between 20% at LOQs and 16% at 1000 μg/L.

Linearity and matrix effects were studied using standard solutions in neat solvent and matrix-matched calibrations. Calibration curves in both pure solvent and matrix were constructed by plotting the analyte relative ion intensity against the concentration at eight concentration levels (from LOQs to 1000 µg/L). Regression coefficients obtained were higher than 0.990 in all cases. In everyday practice the calibration curve was constructed from a single lot of apple matrix. The degree of how SSE might vary in orange juice and pineapple matrixes was also evaluated. The relative errors between the obtained SSEs in the studied matrixes were lower than 18%. Due to the significant values obtained of SSEs, this parameter has been taken account and matrix matched curves were used for quantification.

The here proposed methodology is presented as a sensitive and robust analytical tool for the simultaneous determination of thirty mycotoxins in juice samples.

### 2.2. Mycotoxin Contents in Juices

**Figure 1:**
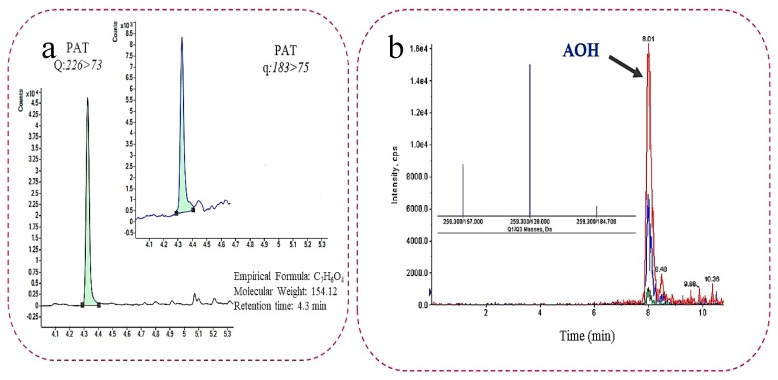
Chromatograms of contaminated samples with (**a**) patulin (PAT) (28.37 µg/L) and (**b**) AOH (441.5 µg/L) determined by GC-MS/MS and LC-MS/MS, respectively.

Only 9 of the 30 analyzed mycotoxins were detected in quantifiable amounts in 49% of the analyzed juices, mainly AOH, AME, PAT, OTA, AFB1, AFB2, AFG2, β-ZAL and HT-2. AOH and PAT, were the most detected mycotoxins with incidences of 29% and 18%, respectively, while β-ZAL and HT-2 were detected in only 3% of the samples (Table 2). The results obtained are consistent with the information available in bibliography, where PAT, AOH and AME are the most common mycotoxins found in fruit juices [2]. Chromatograms of real samples contaminated by PAT at 28.37 µg/L and AOH at 441.5 µg/L, respectively are shown in Figure 1.

Regarding the contents determined, the mean of positive samples ranged from 3.75 to 207.01 µg/L. AOH and PAT were also the mycotoxins detected with the higher contents (Table 2).

The co-occurrence of mycotoxins was found in 9 analyzed samples (12%). Two apple juices showed co-occurrence of two mycotoxins (AOH + β-ZAL and ΒZAL + PAT)**.** The most prevalent co-occurrence was AOH, AME and AFB1, detected simultaneously in 3 samples (2 samples of pineapple juices and one of peach juice) and coexistence of 7 mycotoxins was observed in two samples of fresh orange juice contaminated by AOH, AME, AFB1, AFB2, AFG2, OTA, PAT. Sum of mycotoxins concentrations quantified in the same sample are listed in Table 3. Although the sum of mycotoxins amounts in multi-contaminated samples cannot yet be used to obtain significant interpretations of the risk assessment, the results obtained provide data on the combined and simultaneous exposure to mycotoxins through juice intake. The natural co-occurrence of multiple mycotoxins in food products is an increasing health concern due to the exposure to multiple fungal growths. Very scarce data related to in vivo toxicity evaluation of exposure to combined mycotoxins are available in literature but in line with the in vitro observations, possible additive or synergistic effect may be registered, but, the impact of this effects usually rely on the concentration and duration of exposure [18].

PAT was detected in juices with incidence of 18%, concentrations ranged between < LOQ to 50.95 µg/L and mean of positive samples was about 28.18 µg/L. Only one sample, exceeded slightly the maximum limit set for PAT by the European Commission in fruit juices (50 µg/L) [12]. In Malaysia, Lee et al. [4] found to be PAT positive 5% of 56 analyzed fruit juices in a range of concentration between 13.1 and 33.7 µg/L. In Iran, Rahimi et al. [19] revealed also PAT presence in 16% of 161 fruit juices samples with levels between 5 to 190.7 µg/kg and mean of positives of 34.5 µg/kg. Contrary to the present study, in Tunisia, Zouaoui et al. [20] reported PAT incidence of 50% in 214 samples of fruit juices, compote and jam samples and concentrations ranging from 2 to 889 µg/L and a mean of 89 µg/L. In Pakistan, Iqbal et al. [8] also reported PAT incidence of 57.4% in 237 samples of fruits, juices, and smoothies, with concentrations ranging from 0.04 to 1100 µg/kg.

OTA was determined in 9% of the studied samples at concentrations between 2.93 and 10.81 µg/L. The European Commission has set 2 µg/kg as a limit for OTA on grape juice. In Turkey, Akdeniz et al. [3] found to be positive 20% of 10 grape juice samples with concentrations ranging from 0.9 to 1.9 µg/kg. Asadi et al. [7] also reported lower contents of OTA in 15% of 20 apple juices and 25% of 20 grape juices, ranging from 0.06 to 0.1 µg/L and 0.06 to 0.12 µg/L, respectively.

AME and AOH were detected in 10 and 29% of the analyzed samples at mean concentration of 8.5 and 207 µg/L, respectively. In the study performed by Zwickel et al. [6], AOH and AME were reported at similar incidence (27 and 5% of 78 juice samples) respectively from the German market, similar incidences to the present work, but at lower contents: AOH (from 0.81 to 8.16 µg/L) and AME (from 0.89 to 1.54 µg/L). Myresiotis et al. [5] did not report AOH and AME presence in any of the pomegranate fruit and juice samples purchased from Greek markets, neither Ruan et al. [16] did detect them in commercial fruit juices from the local market of Guangzhou (China).

AFB1, AFB2 and AFG2 were detected in 8, 6 and 4% of the analyzed samples, respectively at levels in the range of 1.24 and 18.1 µg/L. In a study performed in Egypt, Abdel-Sater et al. [21] reported that 100% of five apple beverages resulted positive for AFB1 and AFG1 in a range from 20 to 30 µg/L and two of five guava juices presented AFB1 at 12 µg/L.

Only 2 apple juice samples of 80 samples resulted contaminated by β-ZAL with concentrations of 22.59 and 23.85 µg/L, respectively. Scarce information is available in the literature about the presence of ZEA and its metabolites in fruit juices. In a prior study, Carballo et al. [9] did not find ZEA and β-ZAL presence in a multimycotoxin study performed in 42 samples of fruit juices. Abdel-Sater et al. [21] also found no ZEA presence in fruit juices and beverages. The same case was observed for HT-2, that only was present in 2 samples of mixed fruit juices with concentrations of 21.38 and 24.15 µg/L, respectively. HT-2 is also not commonly found in fruits and its derived products and little information is available in bibliography. López et al. [22] detected HT-2 in apple juice composite but at levels below the LOQ.

Comparing the results per type of juice samples, mono-fruit juice samples presented contamination up to 8 different mycotoxins (AOH, AME, PAT, AFB1, AFB2, AFG2, OTA, β-ZAL) at levels below 50 µg/L, while blended beverages presented contamination by AOH, PAT and HT-2 (Table 4). However, the contents of AOH, the most detected mycotoxin and with the highest levels, were higher in blended beverages, maybe for the addition of other ingredients, like ginger, vegetables as cucumber and spinach or passion fruits. In the case of Ginger, even that its essential oils may inhibit fungal growth, some authors reported ginger contamination by mycotoxins specially OTA and AFs [23]. Vegetables like tomato or red fruits have also been reported contaminated by AOH [24,25]. Juan et al. [26] also reported AOH in strawberry samples up to 752 µg/kg, amounts similar to those reported in the present study. Fresh orange juice samples resulted the most contaminated juice mainly by PAT, AME and AFB2 even at trace levels. AME was previously detected in 11% of citrus juices samples at concentration levels between 0.11 and 0.20 µg/kg [27]. Juan et al. [28] reported AFs, OTA, AOH and AME contamination with mean concentration of 26.12, 0.68, 18.7 and 160.5 µg/L, respectively in berry juices.

### 2.3. Dietary Exposure to Mycotoxins through Fruit Juices Consumption in Children and Adult Population.

With the purpose to evaluate the exposure of children and adult population to mycotoxins through the consumption of fruit juices, the Estimate Daily Intakes (EDIs) were calculated and compared with the Tolerable Daily Intakes (TDIs).

The European Commission has established TDIs for OTA, PAT, and HT2 and βZAL. A provisional maximum tolerable daily intake of 0.4 µg/kg bw/day has been fixed for PAT [29], a TDI of 0.25 µg/kg bw/day for ZEA and of 0.1 µg/kg bw/day for the sum of HT2 and T2 have been set [30]. Tolerable Weekly Intake (TWI) of 0.12 µg/kg bw/week has been also fixed for OTA [31].

The EDIs (μg/kg bw/day) for each mycotoxin were calculated by following formula = C × K/bw where C is the mean concentration of each mycotoxin in juice (µg/L), K is the daily average of juice consumption per person (L/day) and bw is the bodyweight used for the population group.

Left-censored results (data below LOQ) were processed according to EFSA recommendations considering two exposure scenarios [32]. In the lower bound scenario (LB) zero was assigned when mycotoxins were not detected or were detected below the limit of quantification. In the upper bound (UB) scenario, the limit of detection was assigned when mycotoxins were not detected, and the limit of quantification when mycotoxins were detected at levels below LOQ.

A bodyweight of 70 kg was considered for adult population and of 25 kg for children population. The daily consumption of juice extracted from the database of the Spanish Ministry of Agriculture, Fisheries and Food was on average of 9.2 L per year/per person [33]. Children populations are higher consumers of juices and a daily consumption of 200 mL corresponding to single portion was considered for a more realistic approach.

Considering the mean of positive samples as mean concentration, the EDIs obtained represented a notable percentage of TDIs fixed (Table 5). For adult population, percentages of (2.5% TDI), (11.5% TDI), (3.3% TDI) and (8.2% TDI) were respectively obtained for PAT, OTA, βZAL and HT-2. However, when the mean of total samples was used, the EDIs values obtained for adult population were far below the TDIs fixed, representing less than 2% of TDIs, in both LB and UB approaches. 

For children, exposure rate of 56.4% TDI for PAT and 74.3% TDI for βZAL, and EDIs over than the fixed TDI for OTA and HT-2 were obtained with mean of positive samples. This data lowered to 13.8% of PAT TDI, 35.3% of OTA TDI, 9.15% of βZAL TDI and 9% of HT-2 TDI in UB scenario when the mean used was of the whole samples. These results evidenced an increasing risk for children.

Comparing these results with those obtained by Torovic et al. [34] in a study of risk assessment of patulin intake through apple juices in infants and preschool children in Serbia, similar risk exposure was observed by these authors after considering the same daily juice consumption of 0.2 L and the mean of total samples. In this case, the obtained values of EDI for preschool children reached the 6% of the TDI established for PAT, considering different scenarios for left censored results.

## 3. Conclusions

The analytical procedure employed was suitable for the analysis of 30 mycotoxins in the studied juice samples. The mycotoxins determined in the present study such as AOH, AME, and PAT were the most commonly reported in the literature. The main mycotoxins incidences and means in positive samples were lower than 30% and 28.18 µg/L, respectively. AOH was related to the higher mean detected (207 µg/L). A total of 12% of analyzed samples presented coexistence from 2 mycotoxins to 7 mycotoxins. The EDIs calculated for OTA and HT-2 overlapped the established TDIs when the mean of positives samples was considered for risk assessment for children, decreasing to unconcerned levels when the LB and UB scenarios where employed.

## 4. Materials and Methods 

### 4.1. Reagents and Chemicals

Solvents (acetonitrile, methanol, and chloroform) were supplied by Merck (Darmstadt, Germany). Ethyl acetate was supplied by Alfa Aesar (Karlsruhe, Germany). Deionized water (resistivity > 18 MΩ cm^−1^) was obtained in the laboratory using a Milli-Q SP^®^ Reagent Water System (Millipore Corporation, Bedford, MA, USA). Ammonium formate (99%) was supplied by Panreac Quimica S.A.U. (Barcelona, Spain). Formic acid (reagent grade ≥ 95%) was obtained from Sigma Aldrich (St. Louis, MO, USA). Nylon filters (0.45-μm pore size) were supplied by Scharlau (Barcelona, Spain). Syringe nylon filters (13 mm diameter and 0.22-μm pore size) were obtained from Membrane Solutions (Plano, TX, USA). The derivatization reagent composed of BSA (N,O-bis(trimethylsilyl) acetamide) + TMCS (trimethylchlorosilane) + TMSI (N-trimethylsilylimidazole) (3:2:3) was obtained from Supelco (Bellefonte, PA, USA). Disodium phosphate and Sodium dihydrogen phosphate, used to prepare phosphate buffer, were obtained from Panreac.

The standards of AFB1, AFB2, AFG1, AFG2, AOH, AME, OTA, FB1, FB2, ENNA, ENNA1, ENNB, ENNB1, BEA, STG, ZEA, α-ZAL, β-ZAL, α-ZOL, β-ZOL, DON, 3-ADON, 15-ADON, DAS, NIV, FUS-X, NEO, PAT, T-2 and HT-2 toxins were purchased from Sigma Aldrich. Individual stock solutions were prepared to obtain 100 mg/L in methanol and working solutions were obtained diluting the individual stock solutions. All solutions were stored in darkness and kept at −20 °C.

### 4.2. Sampling

A total of 80 commercial juices were collected from various supermarkets of Valencia (Spain) during 2018. The samples were classified into two groups. 40 were mono-fruit; 7 were filtered orange juices with nothing added, and 33 resulting from concentrated, sweetened drinks made from fruit pulpe, where 3 were from orange, 4 from pear, 12 from apple and 7 were from pineapple and peach respectively.

40 blended beverages, containing fruit juices and mashed vegetables and, sometimes incorporating dairy or functional ingredients (e.g., chia seeds). In this group, 5 samples held ecological label, 24 health claims label and 11 common label. The composition of each sample is detailed in Table S1. The samples were stored in darkness and dry place until analysis.

### 4.3. Dispersive Liquid-Liquid Microextraction Procedure (DLLME)

The samples were extracted according to Carballo et al., [9]. 5 mL of each juice sample were placed with 1 g of NaCl in a 10 mL conical tube, and the tube was shaken in vortex for one minute. After vortexing, a mixture of dispersion solvent (950 μL of acetonitrile) and extraction solvent (620 μL of ethyl acetate) was added and the tube was shaken in vortex for one minute, resulting in a cloudy solution of the three components. Then the mixture was centrifuged at 4000 rpm for 5 min, and 600 μL of the organic phase located at the top of the tube was separated and placed into other conical tube. Next, in a second step the mixture of dispersion solvent (950 μL of methanol) and extraction solvent (620 μL of chloroform) was added to the remaining residue in the tube. After centrifugation, 600 μL of the organic phase, located in this case in the bottom of the tube was recovered and added to the first organic phase separated before. Then the two recovered phases were evaporated to near dryness under a nitrogen stream using a Turvovap LV Evaporator (Zymark, Hoptikinton, MA, USA). 

For LC-MS/MS analysis, the residue was reconstituted with 1 mL of 20 mM ammonium formate (MeOH/ACN) (50/50 v/v) and filtered through a 13 mm/0.22 μm nylon filter in a vial while for GC-MS/MS analysis, a derivatization process was carried out by adding 50 μL of BSA + TMCS + TMSI (3:2:3) to the dry extract and left at room temperature 30 min. Then 200 μL of hexane was added, mixed in vortex for 30 s, washed with 1 mL of phosphate buffer (60 mM, pH7) and mixed until the upper layer was clear. Finally, the hexane layer (200 µL) was transferred to an auto sampler vial.

### 4.4. LC-MS/MS Determination

The determination was performed using an Agilent 1200 chromatograph (Agilent Technologies, Palo Alto, CA, USA) equipped with 3200 QTRAP^®^ (Applied Biosystems, AB Sciex, Foster City, CA, USA) with turbo ion spray electrospray ionization (ESI). The QTRAP analyser combines a fully functional triple quadrupole and a linear ion trap mass spectrometer. The chromatographic separation of analytes was performed in a Gemini-NX column C_18_ (Phenomenex, 150 mm × 4.6 mm, 5 particle size) preceded by a guard column. Mobile phases were 5 mM ammonium formate, 0.1% formic acid in water (mobile phase A) and 0.1% formic acid in methanol (mobile phase B). The elution gradient initiated with a proportion of 0% for eluent B; in 10 min increased to 100%, then decreased to 80% in 5 min, and finally decreased to 70% in 2 min. In the next 6 min, the column was cleaned, readjusted to initial conditions, and equilibrated for 7 min. The flow rate was established at 0.25 mL/min, and the oven temperature at 40 °C.

The analysis was performed using the Turbo-ion spray in the positive ionization mode (ESI+). Nitrogen was served as nebulizer and collision gas. During the analysis, the following parameters were fixed: probe temperature 450 °C; ion spray voltage 5500 V; curtain gas 20 arbitrary units; GS1 and GS2, 50 and 50 psi, respectively.

The transitions used for the quantification and confirmation of the monitored fragments are shown in Table 1.

### 4.5. GC-MS/MS Determination 

An GC system Agilent 7890A coupled with an Agilent 7000A triple quadruple mass spectrometer with inter electron-impact ion source (EI, 70Ev) and Agilent 7693 auto sampler (Agilent Technologies, Palo Alto, USA) was used for the determination. Quantitation data were acquired at selection reaction monitoring mode (SRM). The transfer line and source temperatures were 280° and 230°, respectively. Nitrogen was used as collision gas for MS/MS experiments, and helium was used as quenching gas, both at 99.999% purity supplied by Carburos Metálicos S.L. (Barcelona, Spain). A capillary column HP-5MS 30m × 0.25mm × 0.25μm was used for the separation of analytes. One microliter of the final clean derivatized extract of mycotoxins was injected in splitless mode in the programmable temperature vaporization (PTV) inlet at 250 °C, employing helium as carried gas at fixed pressure of 20.3 psi. Oven temperature started at 80 °C, and increased to 245 °C at 60 °C/min, hold their time for 3 min and progressively increased to 260 °C by 3 °C/min and finally to 270 °C at 10 °C/min and then held for 10 min. Agilent Mass hunter version B.04.00 software was employed to acquire and process data. 

## Figures and Tables

**Table 1:** Quantification and confirmation transitions of mycotoxins monitored fragments, retention time (Rt)) and analytical parameters obtained.

Mycotoxin	Quantification Transition	Confirmation Transition	Rt	SSE (%) ^c^	LOD	LOQ	Recovery (%)		
							50	100	200
	m/z	m/z	min		µg/L	µg/L	µg/L	µg/L	µg/L
ZEA ^a^	462 > 151	462 > 333	15.95	115	2.34	7.81	98	113	103
α-ZAL ^a^	433 > 309	433 > 295	15.45	88	0.58	1.95	101	97	99
β-ZAL ^a^	433 > 295	307 > 73	15.68	91	2.34	7.81	97	105	104
α-ZOL ^a^	305 > 73	305 > 289	16.45	62	0.58	1.95	80	113	95
β-ZOL ^a^	536 > 333	536 > 446	16.82	69	1.17	3.90	98	102	69
DON ^a^	392 > 259	407 > 197	8.4	103	0.58	1.95	68	72	63
3-ADON ^a^	392 > 287	467 > 147	9.68	88	1.17	3.90	103	103	105
15-ADON ^a^	392 > 217	392 > 184	9.65	114	0.58	1.95	95	98	96
DAS ^a^	350 > 229	378 > 124	9.85	96	1.17	3.90	103	91	84
NIV ^a^	289 > 73	379 > 73	10.15	87	1.17	3.90	61	88	97
FUS-X ^a^	450 > 260	450 > 245	9.73	106	2.34	7.81	84	98	106
NEO ^a^	252 > 195	252 > 167	11.68	95	0.58	1.95	83	71	69
PAT ^a^	226 > 73	183 > 75	4.3	78	2.34	7.81	61	96	92
T-2 ^a^	350 > 259	350 > 229	14.8	71	2.34	7.81	98	96	71
HT-2 ^a^	347 > 157	347 > 185	14.39	78	0.58	1.95	106	107	78
AFB1 ^b^	313 > 285	313 > 241	7.41	48	0.3	1.0	111	64	115
AFB2 ^b^	315 > 287	315 > 259	7.36	80	0.3	1.0	83	71	69
AFG1 ^b^	329 > 243	329 > 311	7.23	60	0.3	1.0	63	76	83
AFG2 ^b^	331 > 313	331 > 245	7.13	41	0.3	1.0	79	70	92
AOH ^b^	259 ˃ 128	258 ˃ 184	8.01	67	0.3	1.0	105	114	90
AME ^b^	273 ˃ 128	273 ˃ 228	9.10	120	0.3	1.0	78	89	71
OTA ^b^	404 > 239	404 > 102	8.68	64	0.3	1.0	90	73	71
FB_1_^b^	722 ˃ 334	722 ˃ 352	7.7	75	1.5	5.0	65	86	82
FB_2_^b^	706 ˃ 336	706 ˃ 318	7.85	67	1.5	5.0	75	94	79
ENNA ^b^	699 > 210	699 > 228	11.74	73	0.3	1.0	96	67	78
ENNA1 ^b^	685 > 210	685 > 214	11.3	60	0.3	1.0	109	66	87
ENNB ^b^	657 > 196	657 > 214	10.73	66	0.15	0.5	104	66	85
ENNB1 ^b^	671 > 214	671 > 228	10.68	54	0.3	1.0	110	67	81
BEA ^b^	801 > 784	801 > 244	10.84	52	1.5	5.0	112	65	82
STG ^b^	325 > 281	325 > 310	9.08	106	1.5	5.0	62	66	71

^a^ GC-MS/MS determination. ^b^ LC-MS/MS determination. ^c^ SSE: Signal Suppression-Enhancer.

**Table 2:** Mycotoxins ranges and means found in the analyzed samples (μg/L).

MYCOTOXIN	Mean of Positive Samples	Minimum Concentration	Maximum Concentration	Incidence (%)	Lower Bound Scenario ^a^	Upper Bound Scenario ^b^
AFB1	9.36	4.68	18.1	4	0.35	0.64
AFB2	4.49	1.38	12.49	6	0.28	0.56
AFG2	3.75	1.24	7.60	4	0.14	0.43
AME	8.54	2.47	15.18	10	0.85	1.124
AOH	207	< LOQ	1213	29	59.50	59.73
βZAL	23.22	22.59	23.85	3	0.58	2.86
HT2	22.76	21.38	24.15	3	0.57	1.13
OTA	5.43	2.93	10.81	9	0.48	0.75
PAT	28.18	< LOQ	50.95	18	4.93	6.9

^a^ Lower bound scenario: Mean assigning zero to not detected mycotoxins or mycotoxins detected below their corresponding LOQ. ^b^ Upper bound scenario: Mean assigning the LOD to not detected mycotoxins, and the corresponding LOQ to mycotoxins detected at levels below LOQ.

**Table 3:** Sum of mycotoxins concentrations in multi-contaminated samples (µg/L).

Mycotoxins Co-occurrence	Number of Samples	Sum of Concentrations
AOH + β-ZAL (apple juice)	1	24.86
ΒZAL + PAT (apple juice)	1	50.96
AOH + AME+AFB1 (pineapple juice, peach juice)	3	9.36–36.2
AME + AFB2 + AFG2 + OTA + PAT (fresh orange juice)	1	33.3
AME + AFB1 + AFB2 + OTA + PAT (fresh orange juice)	1	71.33
AOH + AME + AFB1 + AFB2 + AFG2 + OTA + PAT (fresh orange juice)	2	65.47–97.36

**Table 4:** Mycotoxin incidence, concentration range and mean of positive samples (µg/L).

Mycotoxin	Mono-Fruit Juices (n = 40)						Blended Beverages (n = 40)								
	Fresh Orange Juices (n = 7)			Packed Juices (n = 33)			Ecological Label (n = 5)			Health Claims Label (n = 24)			Common Label (n = 11)		
	I ^a^	Mean ± SD µg/L	Range µg/L	I	Mean ± SD µg/L	Range µg/L	I	Mean ± SD µg/L	Range µg/L	I	Mean ± SD µg/L	Range µg/L	I	Mean ± SD µg/L	Range µg/L
AFB1	3/7	9.37 ± 3.03	5.93–11.65	3/33	9.77 ± 7.3	4.68–18.1	nd	---	--	nd	--	--	nd	--	--
AFB2	5/7	4.49 ± 4.53	1.38–12.49	nd	---	--	nd	---	--	nd	---	--	nd	---	--
AFG2	3/7	3.76 ± 3.4	1.24–7.6	nd	---	--	nd	---	--	nd	---	--	nd	---	--
AME	5/7	9.53 ± 2.05	7.03–11.77	3/33	6.87 ± 7.2	2.47–15.18	nd	---	--	nd	---	--	nd	---	--
AOH	2/7	3.59 ± 3.14	1.37–5.81	11/33	3.71 ± 3.55	<LOQ–11.16	2/5	629.74 ± 266	441.5–817.98	8/24	528.01 ± 362.9	93.5–1213.87	nd	---	--
Β-ZAL	nd ^b^	---	--	2/33	23.22 ± 0.89	22.59–23.85	nd	---	--	nd	---	--	nd	---	--
HT2	nd	---	--	nd	---	--	nd	---	--	nd	---	--	2/11	22.77 ± 1.95	21.38–24.15
OTA	5/7	6.25 ± 3.93	2.93–10.81	2/33	3.4 ± 0.38	3.13–3.67	nd	---	--	nd	---	--	nd	---	--
PAT	7/7	34.59 ± 13.9	14.78–50.59	5/33	28.34 ± 14	8.05–47.82	nd	---	--	nd	---	--	2/11	10.89 ± 0	<LOQ–10.89

^a^ Incidence (number positive samples/number total samples). ^b^ not detected.

**Table 5:** Mycotoxin risk assessment through fruit juices consumption for children and adult population.

Mycotoxin	TDI (ng/kg bw)	Positive Samples				Lower Bound Scenario				Upper Bound Scenario			
		EDI (ng/kg bw)		%TDI		EDI (ng/kg bw)		%TDI		EDI (ng/kg bw)		%TDI	
		Children	Adults	Children	Adults	Children	Adults	Children	Adults	Children	Adults	Children	Adults
PAT	400	225.44	10.15	56.36	2.54	39.20	1.76	9.80	0.44	55.20	2.48	13.80	0.62
OTA	17	43.44	1.96	255.53	11.50	3.84	0.17	22.59	1.02	6.00	0.27	35.29	1.59
B-ZAL	250	185.76	8.36	74.30	3.34	4.64	0.21	1.86	0.08	22.88	1.03	9.15	0.41
HT-2	100	182.08	8.20	182.08	8.20	4.56	0.21	4.56	0.21	9.04	0.41	9.04	0.41

## Supplementary Materials

The following are available online at https://www.mdpi.com/2072-6651/11/12/684/s1, Table S1: Description of the analyzed samples.

## References

[1] Mandappa I.M., Basavaraj K., Manonmani H.K. (2018). Analysis of Mycotoxins in Fruit Juices. Fruit Juices.

[2] Fernández-Cruz M.L., Mansilla M.L., Tadeo J.L. (2010). Mycotoxins in fruits and their processed products: Analysis, occurrence and health implications. J. Adv. Res..

[3] Akdeniz A.S., Ozden S., Alpertunga B. (2013). Ochratoxin A in dried grapes and grape-derived products in Turkey. Food Addit. Contam. B.

[4] Lee T.P., Sakai R., Manaf N.A., Rodhi A.M., Saad B. (2014). High performance liquid chromatography method for the determination of patulin and 5-hydroxymethylfurfural in fruit juices marketed in Malaysia. Food Control.

[5] Myresiotis C.K., Testempasis S., Vryzas Z., Karaoglanidis G.S., Papadopoulou-Mourkidou E. (2015). Determination of mycotoxins in pomegranate fruits and juices using a QuEChERS-based method. Food Chem..

[6] Zwickel T., Klaffke H., Richards K., Rychlik M. (2016). Development of a high performance liquid chromatography tandem mass spectrometry based analysis for the simultaneous quantification of various Alternaria toxins in wine, vegetable juices and fruit juices. J. Chromatogr. A.

[7] Asadi M. (2018). Determination of ochratoxin A in fruit juice by high-performance liquid chromatography after vortex-assisted emulsification microextraction based on solidification of floating organic drop. Mycotoxin Res..

[8] Iqbal S.Z., Malik S., Asi M.R., Selamat J., Malik N. (2018). Natural occurrence of patulin in different fruits, juices and smoothies and evaluation of dietary intake in Punjab, Pakistan. Food Control.

[9] Carballo D., Pinheiro-Fernandes-Vieira P., Tolosa J., Font G., Berrada H., Ferrer E. (2018). Dietary exposure to mycotoxins through fruits juice consumption. Rev. Toxicol..

[10] Marin S., Ramos A.J., Cano-Sancho G., Sanchis V. (2013). Mycotoxins: Occurrence, toxicology, and exposure assessment. Food Chem. Toxicol..

[11] Drusch S., Ragab W. (2003). Mycotoxins in fruits, fruit juices, and dried fruits. J. Food Prot..

[12] European Commission (2006). Commission Directive 2006/1881/EC of 19 December 2006, Setting Maximum Levels for Certain Contaminants in Foodstuffs. OJEU.

[13] Escrivá L., Oueslati S., Font G., Manyes L. (2017). Alternaria mycotoxins in food and feed: An overview. J. Food Qual..

[14] Zgoła-Grześkowiak A., Grześkowiak T. (2011). Dispersive liquid-liquid microextraction. TrAC Trends Anal. Chem..

[15] Víctor-Ortega M.D., Lara F.J., García-Campaña A.M., del Olmo-Iruela M. (2013). Evaluation of dispersive liquid–liquid microextraction for the determination of patulin in apple juices using micellar electrokinetic capillary chromatography. Food Control.

[16] Ruan C., Diao X., Zhang H., Zhang L., Liu C. (2016). Development of a dispersive liquid–liquid microextraction technique for the analysis of citrinin, alternariol and alternariol monomethyl ether in fruit juices. Anal. Methods.

[17] European Commission (2002). Decision 2002/657/EC of 12 August 2002, implementing Council Directive 96/23/EC concerning the performance of analytical methods and the interpretation of results. OJEU.

[18] Mallebrera B., Prosperini A., Font G., Ruiz M.J. (2018). In vitro mechanisms of Beauvericin toxicity: A review. Food Chem. Toxicol..

[19] Rahimi E., Rezapoor Jeiran M. (2015). Patulin and its dietary intake by fruit juice consumption in Iran. Food Addit. Contam. B.

[20] Zouaoui N., Sbaii N., Bacha H., Abid-Essefi S. (2015). Occurrence of patulin in various fruit juice marketed in Tunisia. Food Control.

[21] Abdel-Sater M.A., Zohri A.A., Ismail M.A. (2001). Natural contamination of some Egyptian fruit juices and beverages by mycoflora and mycotoxins. J. Food Sci. Tech. MYS.

[22] López P., De Rijk T., Sprong R.C., Mengelers M.J.B., Castenmiller J.J.M., Alewijn M. (2016). A mycotoxin-dedicated total diet study in the Netherlands in 2013: Part II–occurrence. World Mycotoxin J..

[23] Kabak B., Dobson A.D. (2017). Mycotoxins in spices and herbs—An update. Crit. Rev. Food Sci..

[24] Qiao X., Yin J., Yang Y., Zhang J., Shao B., Li H., Chen H. (2018). Determination of Alternaria Mycotoxins in Fresh Sweet Cherries and Cherry-Based Products: Method Validation and Occurrence. J. Agric. Food Chem..

[25] Rodríguez-Carrasco Y., Mañes J., Berrada H., Juan C. (2016). Development and validation of a LC-ESI-MS/MS method for the determination of Alternaria toxins alternariol, alternariol methyl-ether and tentoxin in tomato and tomato-based products. Toxins.

[26] Juan C., Oueslati S., Mañes J. (2016). Evaluation of Alternaria mycotoxins in strawberries: Quantification and storage condition. Food Addit. Contam. A.

[27] Zhao K., Shao B., Yang D., Li F. (2014). Natural occurrence of four Alternaria mycotoxins in tomato-and citrus-based foods in China. J. Agric. Food Chem..

[28] Juan C., Mañes J., Font G., Juan-García A. (2017). Determination of mycotoxins in fruit berry by-products using QuEChERS extraction method. LWT.

[29] European Food Safety Authority (EFSA) (2002). Report of Experts Participating in Task 3.2.8. Assessment of Dietary Intake of Patulin by the Population of EU Member States. https://ec.europa.eu/food/sites/food/files/safety/docs/cs_contaminants_catalogue_patulin_3.2.8_en.pdf.

[30] European Food Safety Authority (EFSA) (2014). Scientific Opinion on the Risks for Human and Animal Health Related to the Presence of Modified Forms of Certain Mycotoxins in Food and Feed. EFSA J..

[31] European Food Safety Authority (EFSA) (2006). Opinion of the scientific Panel on Contaminants in the food chain on a request from the commission related to Ochratoxin A in food (Adopted on 4 April 2006). EFSA J..

[32] European Food Safety Authority (EFSA) (2010). Management of left-censored data in dietary exposure assessment of chemical substances. EFSA J..

[33] Ministry of Agriculture, Fisheries and Food (MAPAMA) (2017). Data on Food Consumption, Fruit Juices. Statistics Database-Home Consumption.

[34] Torović L., Dimitrov N., Assunção R., Alvito P. (2017). Risk assessment of patulin intake through apple-based food by infants and preschool children in Serbia. Addit. Contam. A.

