# Supplementary material for: Mycotoxin Dietary Exposure Assessment through Fruit Juices Consumption in Children and Adult Population
Source: Toxins (Basel). 2019 Nov 22;11(12):684. doi: 10.3390/toxins11120684 (PMC6950291; doi:10.3390/toxins11120684)
Supplement: Supplementary file 1 [file toxins-11-00684-s001.pdf]

Supplementary Materials: Mycotoxin Dietary Exposure Assessment through Fruit Juices Consumption in Children and Adult Population

Noelia Pallarés, Dionisia Carballo, Emilia Ferrer, Mónica Fernández-Franzón and Houda Berrada

Table S1. Description of the analyzed samples.

**Mono-fruit Juices (n = 40)**		**Blended Beverages (n = 40)**		
**Fresh Juices (n = 7)**	**Packed Juices (n = 33)**	**Ecological Label (n = 5)**	**Health Claims Label (n = 24)**	**Common Label (n = 11)**
			**Containing herbs and spices**	
-Orange (n = 7)	-Orange (n = 3)	-Apple, mango, banana, passion fruit	-Apple, mango, tomato, lemon, beetroot, pepper, goji berries, pomegranate	-Pear, apple, pineapple, lemon
	-Apple (n = 12)	-Apple, strawberry, raspberry, blueberries, banana	-Pumpkin, mango, carrot, passion fruit, ginger, chili extract	-Apple, pear, raspberry, strawberry, beetroot
	-Peach (n = 7)	-Apple, pineapple, banana, coconut milk	-Apple, parsnip, celery, cucumber, Matcha tea, safflower	-Apple, grape, carrot, mango, lemon, passion fruit
	-Pear (n = 4)	-Apple, cucumber, celery, kale, spinach, lemon, ginger	-Apple, carrot, beetroot, lemon, ginger	-Apple, carrot, beetroot, grape, lemon, strawberry, raspberry, blueberries
	-Pineapple (n = 7)	-Carrot, apple, orange, ginger, lemon	-Apple, pumpkin, carrot, pineapple, lemon, ginger, cinnamon	-Pineapple, orange, apple, mango, passion fruit, grape, banana, pear, peach
			-Apple, spinach, celery, lemon, fennel, kiwi	-Apple, pineapple, mango, milk (n = 6)
			-Apple, cucumber, kale, spinach, lemon, ginger, spirulina, lettuce	
			-Apple, spinach, celery, ginger, lemon	
			-Apple, Chia seeds, mango, banana, passion fruit	
			-Apple, pear, banana, cinnamon, yogurt, oats	
			-Apple, carrot, passion fruit, ginger, lemon, ginseng	
			-Apple, banana, pear, mango, spices	
			-Pineapple, apple, banana, pineapple, orange, coconut, lemon, valerian	
			-Apple, orange, aloe vera, raspberry, blueberries, strawberry, pomegranate	
			-Apple, pear, grape, ginger, lemon, spinach, artichoke, sprirulina	
			**Fiber rich**	
			-Orange, apple, carrot, pineapple, pumpkin, citric fiber, guarana, agave syrup	
			-Strawberry, banana, apple, beetroot, raspberry, citric fibre, pea protein, agavae syrup	

-Apple, pineapple, mango, banana, lettuce, coconut, carrot, corn, kiwi, citric fibre, hemp seeds
-Apple, beetroot, pineapple, lemon, ginger, citric fibre
**Antioxidant rich**
-Apple, carrot, peach, pumpkin, ginger, vitamin C
-Apple, carrot, strawberry, beetroot, vitamin C
-Orange, carrot, lemon, vitamin C, E, β-carotene
-Apple, carrot, beetroot, lemon, vitamin C, E
